# Vacunas para proteger a nuestros mayores: eficiencia, responsabilidad y ética

**DOI:** 10.1016/j.aprim.2023.102796

**Published:** 2023-11-09

**Authors:** Sofía Bauer, Javier Díez-Domingo, Laura Vallejo

**Affiliations:** a Medicina Familiar y Comunitaria, Centro de Salud de Malvarrosa; Miembro del grupo de investigación en Atención Primaria en INCLIVA, Valencia, España; b Área de Investigación en Vacunas, FISABIO, Valencia, España; c Acceso al Mercado y Farmacoeconomía, GSK, España

*Sr. Editor,*

Los profesionales de la atención primaria deben perseguir la mejor eficiencia para mejorar la salud ciudadana. Es evidente que la atención primaria se encuentra actualmente en una situación crítica debido a la precariedad laboral, la excesiva burocracia y la escasa financiación. Esto se traduce en una sobrecarga asistencial que dificulta la entrega de una atención de calidad a los pacientes.

En este contexto, es fundamental centrarse en las actividades de prevención primaria y de promoción de la salud como una manera de mejorar la calidad de vida de la población. Debe buscarse la eficiencia en las decisiones clínicas y enfocarse en las intervenciones que aporten mayor beneficio en términos de salud pública con la mejor ratio coste/beneficio posible.

La población española está envejeciendo, lo que se asocia con una *inmunosenescencia*, más marcada cuando existen enfermedades crónicas, así como con un incremento en la incidencia de enfermedades infecciosas, algunas de las cuales pueden ser prevenidas mediante la vacunación^1^.

Las enfermedades producidas por el neumococo, la gripe, la tosferina y el herpes zóster (HZ) son responsables de aproximadamente el 94% de la carga que provocan enfermedades prevenibles por vacunas en adultos. Las consecuencias económicas no son nada desdeñables: las hospitalizaciones son el principal determinante de los costes directos para la gripe y la enfermedad neumocócica; sin embargo, en el HZ, ese papel lo ocupan las visitas al médico de familia^2^. Además, las pérdidas en calidad de vida son de magnitud relevante^3^, similar o superior, a la observada en otras enfermedades como la artritis reumatoide o la espondilitis anquilosante, por citar algunos ejemplos.

Existe un número creciente de vacunas en desarrollo que van a mejorar la salud de la población mayor. Algunas han sido incorporadas recientemente al calendario vacunal (como HZ), y es de esperar que otras lo hagan próximamente dado su impacto poblacional (como el virus respiratorio sincitial). Sin embargo, a pesar de que las vacunas son la medida más coste/efectiva para mejorar la salud pública, las coberturas vacunales para adultos mayores de 65 años están muy lejos de las infantiles. Algunas reticencias a recomendar vacunas están basadas en análisis parciales de la evidencia, lejos de los dictámenes de la ponencia de salud pública. En este sentido, su seguridad es siempre una de las prioridades de las autoridades regulatorias, que solo aprobarán su uso una vez esté demostrada la gran relación beneficio/riesgo. Por otro lado, no administrar las vacunas recomendadas y financiadas por el sistema sanitario supone dejar vulnerables a los pacientes, pudiendo ser cuestionado desde un punto de vista ético.

En conclusión, creemos que se debe promover la vacunación como una medida preventiva eficiente frente a enfermedades infecciosas potencialmente graves y especialmente en las personas mayores, que son las más vulnerables a estas enfermedades. Los médicos de atención primaria deben seguir las recomendaciones al respecto para garantizar la salud y el bienestar de los pacientes.

## **Conflicto de intereses**

Sofía Bauer Izquierdo es miembro del Grupo de Investigación en Atención Primaria del INCLIVA, Javier Díez-Domingo es jefe del Área de Investigación en Vacunas de FISABIO y Laura Vallejo trabaja para el Laboratorio GlaxoSmithKline (GSK).
